# The Effects of Aromatherapy on Anxiety and Depression in People With Cancer: A Systematic Review and Meta-Analysis

**DOI:** 10.3389/fpubh.2022.853056

**Published:** 2022-05-30

**Authors:** Dan Li, Yuxin Li, Xue Bai, Meijuan Wang, Jingzheng Yan, Yingjuan Cao

**Affiliations:** ^1^School of Nursing and Rehabilitation, Cheeloo College of Medicine, Shandong University, Jinan, China; ^2^Department of Nursing, Qilu Hospital, Cheeloo College of Medicine, Shandong University, Jinan, China; ^3^Nursing Theory and Practice Innovation Research Center, Shandong University, Jinan, China; ^4^Department of Nursing and Health Management, Baotou Iron and Steel Vocational and Technical College, Baotou, China

**Keywords:** aromatherapy, anxiety, depression, cancer, systematic review, meta-analysis

## Abstract

**Background:**

Anxiety and depression are highly prevalent in people with cancer. Medical therapies are usually prescribed to alleviate anxiety and depression, but they are associated with a variety of adverse effects. Recently, aromatherapy showed potential as a complementary medicine to improve psychological health and wellbeing. However, its effectiveness on relieving anxiety and depression has not been established.

**Objective:**

This study explored the beneficial effects of aromatherapy on psychological symptoms such as anxiety and depression in people with cancer.

**Methods:**

We searched international databases including PubMed, Web of Science, Cochrane Library, Embase, Medline, Ebscohost, ProQuest and Scopus from inception to 31 May 2021. The risk of bias was assessed using the Cochrane Collaboration's tool for assessing risk of bias. The systematic review and meta-analysis were performed according to the PRISMA guidelines. Quantitative analysis was performed on the studies that met our inclusion criteria, and Meta-analysis was performed on the studies with available data by RevMan software.

**Results:**

The quality of the literatures were assessed carefully by two researchers, a total of 17 studies were included in the systematic review and 10 articles were conducted in meta-analysis. The aromatherapy was effective in relieving anxiety (SMD = −0.49, *p* < 0.05) in people with cancer. Subgroup analysis suggested that most effective methods were aromatic massage (*SMD* = −0.70, *p* < 0.005), aromatherapy with lavender essential oils (*SMD* = −1.12, *p* < 0.01), short-time interventions (duration < 4weeks) (*SMD* = −0.87, *p* < 0.05) and studies in Asia (*SMD* = −0.83, *p* < 0.05). Regarding depression and psychological wellbeing, there were no difference between aromatherapy and control groups.

**Conclusion:**

In cancer patients, the aromatherapy was effective for relieving anxiety. However, there was no beneficial effect on depression and psychological wellbeing.

**Systematic Review Registration:**

PROSPERO, identifier: *CRD*42021272465.

## Introduction

People with cancer may experience a variety of physical and psychological symptoms (1–3). Anxiety and depression are relatively common and have negative effects on the quality of life (4, 5). The patients with untreated anxiety or depression are less likely to take treatment medications, or may withdraw from family or other social support systems (6–9). Nowadays, a continuous screening for anxiety and depression with proper treatment interventions is recommended for a good cancer care (10, 11). Different drugs are used to treat anxiety and depression, but they are associated with a number of adverse events such as headache, confusion, seizures and addiction (12, 13). The use of complementary and alternative medicine (CAM) is becoming increasingly common among cancer patients who suffer from anxiety and depression, including chiropractic and relaxation exercises (14), music therapy (15), acupuncture, reflexology and aromatherapy (16–18).

The aromatherapy is based on the usage of essential oils extracted from natural plants to promote health and wellbeing (19). Main molecules of essential oils are inhaled with various methods or absorbed through the external skin during massage therapy. Aromatherapy massage using essential oils is recognized as a mind-body therapy that works primarily on the nervous system, but may also stimulate the immune system and affect emotions (20). Inhalation aromatherapy works through the stimulation of the olfactory receptors in the olfactory bulb, which transmit signals to the limbic system and hypothalamus, where the brain secretes neurotransmitters such as serotonin and dopamine which further eased psychological problems (21). Although the exact mechanism is unclear, aromatherapy has been widely used for its ease of use, safety and convenience (22, 23). The effectiveness of aromatherapy has been demonstrated in lower and upper respiratory infections (24), sleep disorders (25), nausea, vomiting (26) and pain (27, 28). A systematic review evaluated the effects of massage with or without aromatherapy on symptoms relief in people with cancer. However, this review only focused on massage aromatherapy, while other delivery methods were not evaluated. Furthermore, the review included only 2 randomized controlled trials conducted in 1999 and 2004 to explore the effect on anxiety. There were too few included studies to be reliable and key outcomes were not reported (29). Therefore, the aim of this study was to systematically review and meta-analyse studies investigating the effects of aromatherapy interventions on anxiety and depression for people with cancer.

## Materials and Methods

### Study Design

The study was conducted following the Preferred Reporting Items for Systematic Reviews and Meta-Analyses (PRISMA) guidelines. The study protocol was registered on PROSPERO (CRD42021272465).

### Searching Terms

We searched for randomized controlled trials investigating the effects of aromatherapy interventions on anxiety, depression or psychology in people with cancer. The PICO principle was adopted to screen articles and the searching terms were utilized as following:

#### Population

The study population included people with a diagnosis of cancer. There were no restrictions on the type of cancer studied.

#### Intervention

The aromatherapy interventions consisted of inhalation and/or massage therapy with essential oils. There were no restrictions on the type of essential oils administered.

#### Control

The control interventions could be placebo, routine care or other methods.

#### Outcomes

We included studies measuring the effects of aromatherapy on anxiety, depression or psychological wellbeing. Outcomes were measured using validated tools.

The exclusion criteria were: (1) overlapping studies, (2) articles not published in English, (3) case reports, cohort studies, qualitative studies, reviews, conference articles, animal researches and study protocols of randomized controlled trials, (4) not full-text articles.

### Search Strategies

We searched international databases including Pubmed, Web of Science, Cochrane Library, Embase, Medline, Ebscohost, ProQuest and Scopus from inception to 31 May 2021. We combined medical subject headings terms and free-text terms to identify eligible studies. The keywords included aromatherapy, essential oil, fragrance, anxiety, anxious, depression, emotion, psychology, cancer, tumor, and neoplasm. The searching strategy was independently conducted by two researchers (LD and LYX) and adapted for differences across databases. The Pubmed searching strategy was displayed in Table 1. The search strategies of other databases were displayed in Supplementary Table 2.

**Table 1:** Searching strategy in pubmed.

**No**.	**Searching term**
1	“aromatherapy” [Mesh]
2	aromatherapy* [Title/Abstract]
3	fragrance [Title/Abstract]
4	essential oil [Title/Abstract]
5	scent therapy [Title/Abstract]
6	aroma therapy [Title/Abstract]
7	1 OR 2 OR 3 OR 4 OR 5 OR 6
8	“anxiety” [Mesh]
9	“depression” [Mesh]
10	anxi* [Title/Abstract]
11	depress* [Title/Abstract]
12	emotion* [Title/Abstract]
13	psycholog* [Title/Abstract]
14	disorder* [Title/Abstract]
15	8 OR 9 OR 10 OR 11 OR 12 OR 13 OR 14
16	“neoplasms” [Mesh]
17	neoplas* [Title/Abstract]
18	cancer [Title/Abstract]
19	tumour [Title/Abstract]
20	tumor [Title/Abstract]
21	carcinoma [Title/Abstract]
22	malignancy [Title/Abstract]
23	16 OR 17 OR 18 OR 19 OR 20 OR 21 OR 22
24	7 AND 15 AND 23

### Study Selection

The search results were imported into Noteexpress software. After duplicated studies being removed, two researchers (LD and BX) independently screened the titles and abstracts, removing studies that did not meet the criteria. Then, the full texts were evaluated and studies meeting the criteria were included. Two researchers verified the included studies. In the presence of a different opinion, a third researcher (WMJ) resolved the issue.

### Data Extraction

Two researchers (LD and BX) collected the data. The extracted data were as following: authors, country, number of treatment and control groups, type of aromatherapy, essential oils, duration of aromatherapy, type of control intervention and measurement tools. When the mean and standard deviation were available, we pooled the data to conduct a meta-analysis. If the study was characterized by several treatment groups of aromatherapy, we split the study into different substudies. If the study included different measurement time points, we considered the last one.

### Quality Assessment

Two researchers (LYX and WMJ) independently used the Cochrane Handbook for Systematic Reviews of Interventions to assess the risk of bias. Each study was assessed as follows: the risks of selection bias including random sequence generation and allocation concealment, blinding of participants and personnel, blinding of outcome assessment, incomplete outcome data, selective reporting, and other biases. We regarded other factors that may cause bias provided in the studies as other bias, for example the baseline differences between the control and treatment groups. The risk was evaluated as “low, high, and unclear,” When they had different opinions, a third researcher (YJZ) assisted to resolve issues between them.

### Statistical Analysis

Quantitative meta-analysis was conducted using RevMan software 5.3. A narrative approach was used for studies that could not be quantitatively analyzed in the meta-analysis. A random effects model was used in the meta-analysis. The results were presented by standardized mean difference (SMD) and their 95% confidence interval (95% CI). The overall effect was considered statistically significant when *p* < 0.05. Heterogeneity was assessed by the Higgins I^2^-statistics. The funnel plot was used to evaluate publication bias, with asymmetry representing possible publication bias (30), and verified by Begg's test using Stata 15.0.

## Results

**Figure 1:**
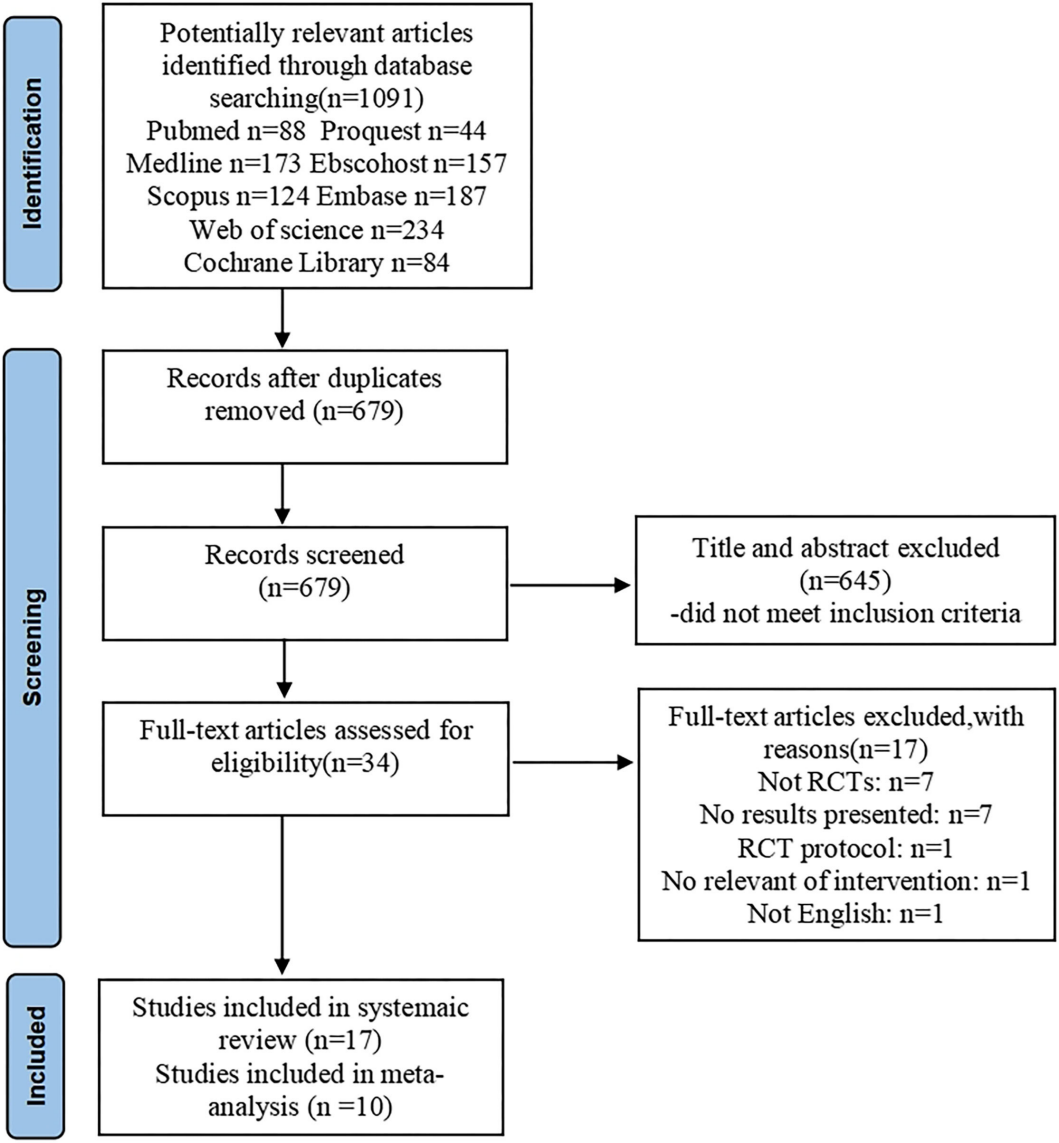
Flowchart of the study.

A total of 1,091 articles were screened. After deleting 412 duplicate studies, two researchers independently reviewed the titles and abstracts of the remaining 679 studies. Of these, 645 studies did not meet the inclusion criteria. The remaining 34 studies were further screened for their final inclusion. A total of 17 studies were excluded for the following reasons: seven studies were not randomized controlled trials, a single study was published not in English, a single study did not use aromatherapy, a single study was a protocol of randomized controlled trial and seven studies did not have full-text articles (Figure 1). We tried to contact the authors for the full articles but did not get a reply. Finally, 17 studies were included in the systematic review (31–47).

### Study Characteristics

The included studies were published between 1995 and 2021. Six studies were conducted in UK, five studies were conducted in Turkey, the other studies were conducted in China, Australia, America, Korea, Brazil and India. All studies were randomized controlled trials, a total of 1,611 people with cancer were enrolled. Nine studies investigated the effects of massage aromatherapy, six studies used inhaled aromatherapy, two studies combined both massage and inhalation. Nine studies utilized a single essential oil, six studies used a mixture of essential oils, two studies did not report specific essential oils. The lavender essential oil was the most frequently used. The duration of aromatherapy session was heterogeneous, while the therapy duration ranged from a single application to 10 weeks. The most common duration of treatment was 4 weeks. Regarding the study of Ozkaraman et al. (40), we used two out of three study subgroups. In two other studies (37, 47), we only extracted data of the outcomes in the final measurements.

Ten studies enrolled cancer patients without limitation of diagnosis (33, 36, 37, 40, 42–47). Two studies enrolled patients with colorectal cancer (31, 35), three studies (32, 34, 39) enrolled patients with breast cancer, one study enrolled patients with gynecological cancer (38) and another study enrolled patients with chronic myeloid leukemia (41).

Fifteen studies evaluated the effects of aromatherapy on anxiety. Different tools were utilized, including the State Anxiety Inventory (SAI) (31, 32, 40, 41, 45–47), the Hospital Anxiety Depression Scale (HADS) (33, 36, 38, 43), the Visual Analog Scale (VAS) (34), the Profile of Mood States (POMS) (37), the Beck Anxiety Inventory (BAI) (42) and the Ostomy Adjustment Inventory (QAI) scale (35). Six studies reported the effects of aromatherapy on depression, utilizing the Center for Epidemiological Studies Depression (CES-D) (47), HADS (33, 36, 38, 43) and POMS (37). In addition, four studies used the Rotterdam Symptom Checklist (RSCL) (39, 43, 45, 46) and one study (44) used the POMS to measure the psychological wellbeing. Table 2 showed the characteristics of each included study.

**Table 2:** Study characteristics.

**Studies**	**Country**	**Intervention (essential oils)**	**Application**	**Control**	**Sample size (n)**	**Duration**	**Scale**
Ayik et al. (31)	Turkey	Lavender oil (Lavandula Hybrida) + almond oil: 5%	Massage	Standard nursing care	80	10 min/day, per 2 days	SAI (+)
Beyliklioglu et al. (32)	Turkey	Lavender oil	Inhalation	Routine care	80	20 min, per 1 day	SAI (+)
Corner et al. (33)	America	A blend of lavender (Lavendula angustifolia), rosewood (Aniba rosaerodora), lemon (Citrus limonum), rose (Rosa damascena) and valerian (Valeriana officinalis) oilsat a ratio of 43:29:17:7:4 (2) carrier oil group: sweet almond oil	Massage	None	52	30 min/week, per 8 weeks	HADS (-)
Deng et al. (34)	China	A blend of lavender, bergamot and geranium oils at a ratio of 1:2:3	Inhalation	Usual care	80	30 min, twice/day	VAS (+)
Duluklu et al. (35)	Turkey	Lavender oil	Inhalation	Routine care	30	15 min/day	OAI-23 (+)
Graham et al. (36)	Australia	A blend of lavender, bergamot and cedarwood oils at a ratio of 2:1:1	Inhalation	(1) fractionated low-grade essential oils (2) pure essential oils (3) Carrier oil	313	15–20 min during radiotherapy	HADS (-)
Noh and Park (38)	Korea	A blend of mandarin, black pepper, pine and tea tree essential oils at a ratio of 1:1:1:1	Massage	None	63	30 min/session, 3 times/week per 6 weeks	HADS (+)
Ovayolu et al. (39)	Turkey	(1) fragrance: a blend of lavender, mint, chamomile, jasmine, violet, rosemary and eucalyptus oils at a ratio of 2:2:2:1:1:1:1 (2) 35 min massage with olive oil (3) 35 min massage with aromatherapy oils	(1) Inhalation (2) Massage (3) Massage	Routine care	280	3 times/week	RSCL-Psychology (+)
Ozkaraman et al. (40)	Turkey	(1) lavender (Lavandula Hybrida) (2) tea tree oil	Inhalation	None	70	5 min/day	SAI (-)
Pimenta et al. (41)	Brazil	(1) 10 mg diazepam (2) Citrus aurantium oil	Inhalation	Saline solution	42	30 min	SAI (+)
Santosh et al. (42)	India	Lavender oil	Massage and Inhalation	None	60	10 min/h, during chemotherapy	BAI (+)
Serfaty et al. (37)	UK	A choice of 20 essential oils	Massage	CBT	36	1 h/session, 8 sessions/10 weeks	POMS (-)
Soden et al. (43)	UK	(1) Lavender oil and an inert carrier oil (2) an inert carrier oil	Massage	None	42	30 min/week, per 4 weeks	HADS (-) RSCL (-)
Wilcock et al. (44)	UK	Lavender + chamomile + sweet almond carrier oil	Massage	Day care	29	30 min/week, per 4 weeks	POMS (-)
Wilkinson et al. (46)	UK	Roman chamomile oil + sweet almond carrier oil	Massage	Carrier oil (sweet almond oil)	46	3 full-body massages per 3 weeks	SAI (-) RSCL (-)
Wilkinson et al. (45)	UK	Roman chamomile oil + sweet almond carrier oil	Massage	Carrier oil (sweet almond oil)	87	3 full-body massages per 3 weeks	SAI (-) RSCL (-)
Wilkinson et al. (47)	UK	A choice of 20 essential oils	Massage	Usual care	221	1 h/week, per 4 weeks	SAI (+) CES-D (-)

### Quality Assessment

**Figure 2:**
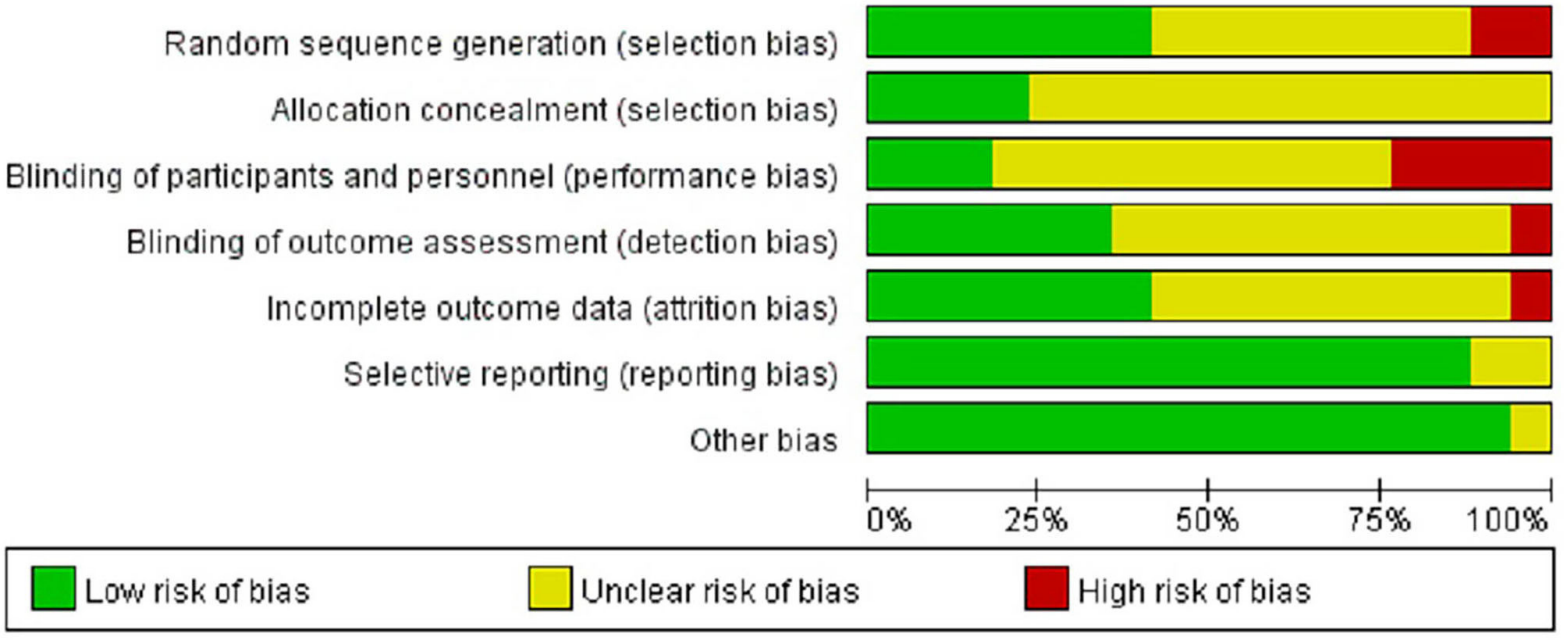
Evaluation of the risk of bias.

**Figure 3:**
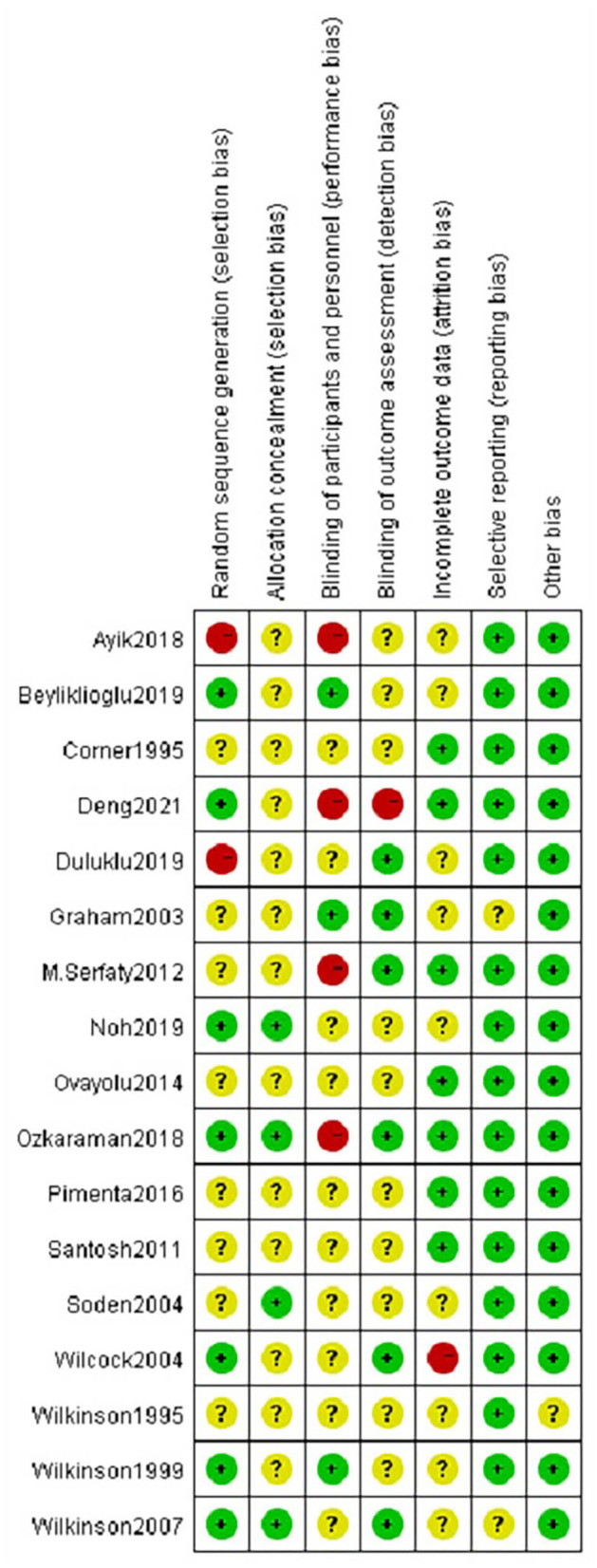
Summary of the risk of bias.

The risk of bias of included studies was presented in Figure 2. Seven studies described the randomization methods. Only 4 out of 17 studies reported using sequential numbered opaque sealed envelopes for allocation concealment. Ten studies did not report the blinding of participants and/or personnel involved in the research, while five studies reported blinding of outcome assessment. No other obvious sources of bias were found in included studies, so the risk of other bias was low. The summary was displayed in Figure 3.

### Study Outcomes

#### Effects of Aromatherapy on Anxiety

**Figure 4:**
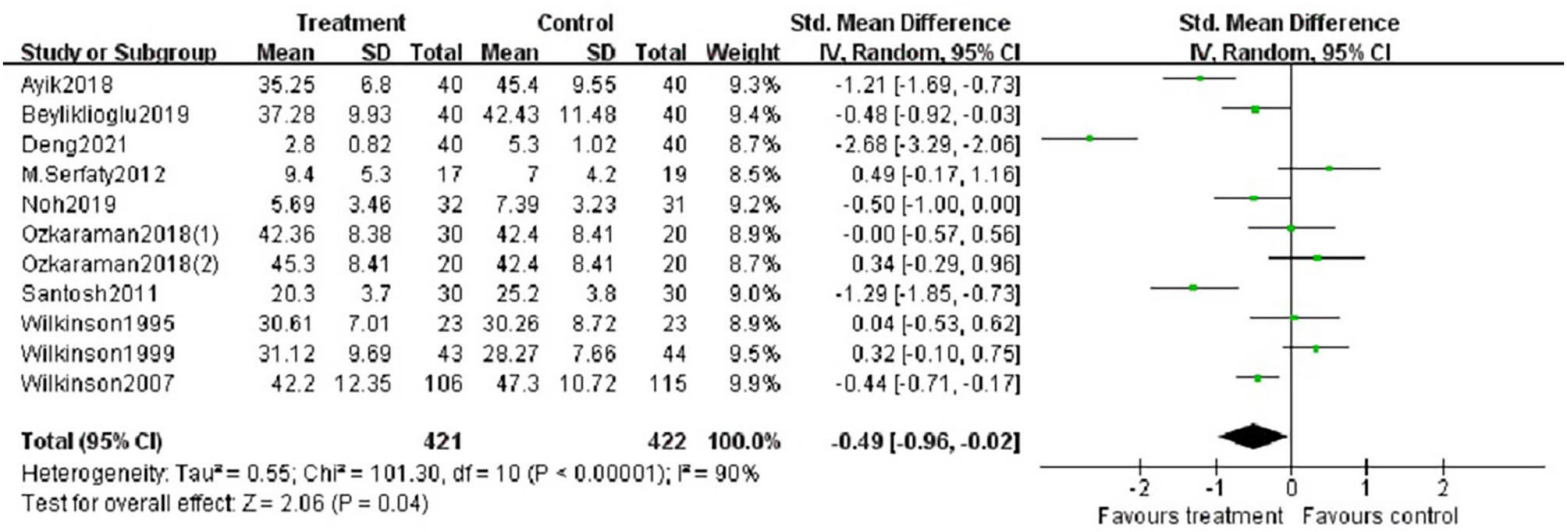
Forest plot showing the pooled effects of aromatherapy on anxiety.

Fifteen studies reported the effects of aromatherapy on anxiety. One study evaluating median change in HADS scores reported no statistically significant changes between the groups (43). Another study reported that massage with essential oils reduced anxiety scores, but not massage with plain oil (33). Graham et al. claimed that the percentage of patients with HADS scores >7 in the fragrant placebo, non-fragrant placebo, and essential oil groups decreased from 26, 25, and 32% at baseline to 23, 13, and 26% at treatment completion, respectively (36). One study using the SAI scores reported that aromatherapy was effective against anxiety (41). Another study reported similar results using OAI scales (35). Ten studies with 11 trials were included in the meta-analysis. The number of participants was 421 patients in the aromatherapy group and 402 patients as controls. According to the random effects model, the average change of anxiety scores between treatment and control groups was −0.49 (95% CI: −0.96– −0.02, Z = 2.06, *p* < 0.05). The I^2^ value was 90%, describing a high heterogeneity among studies (Figure 4).

##### Publication Bias and Sensitivity Analysis

**Figure 5:**
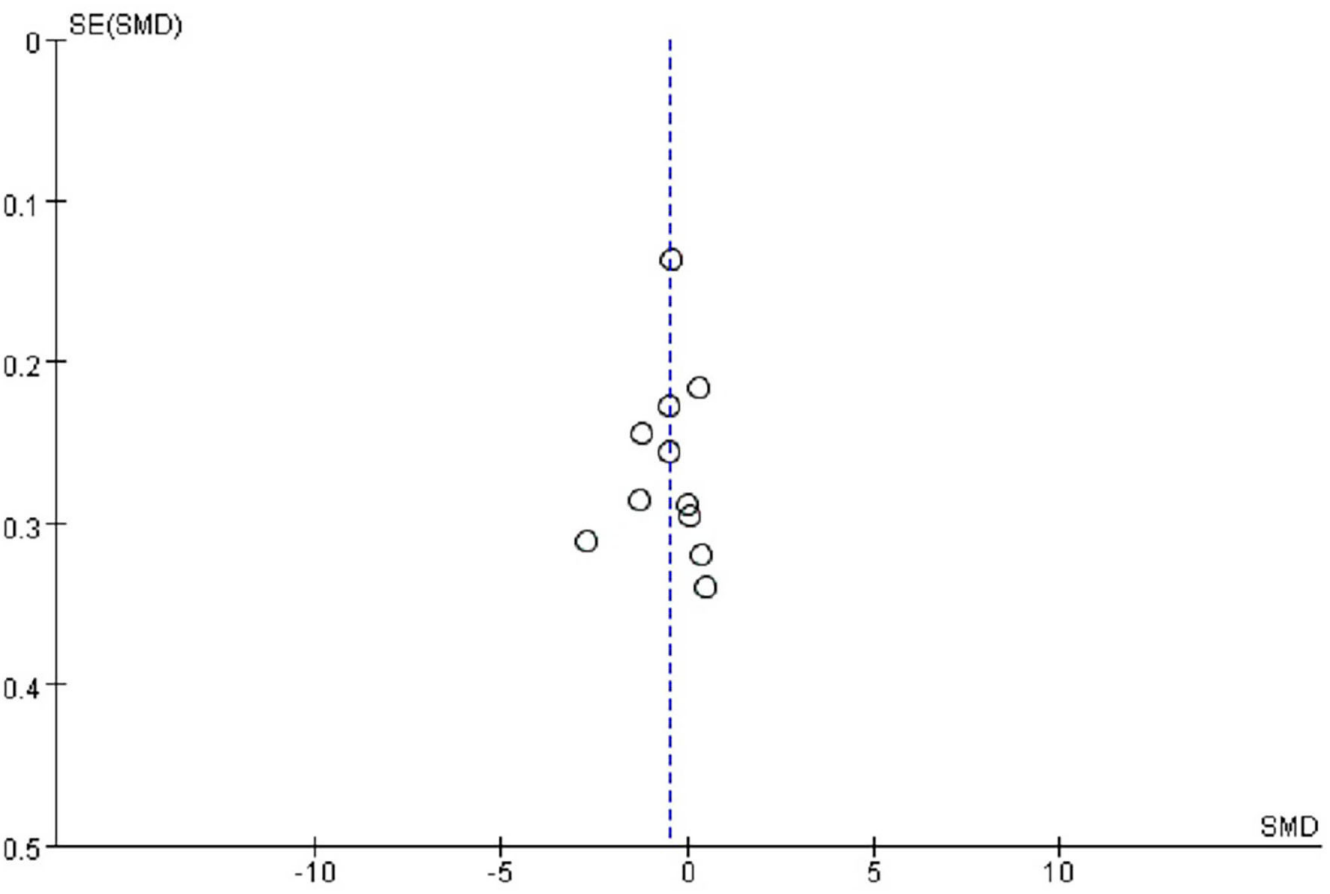
Funnel plot for publication bias.

As displayed in Figure 5, no obvious asymmetry appeared in the funnel plot, and Begg's test revealed no publication bias (*t* = 0.47, *p* = 0.640). Considering the high heterogeneity of the included studies, sensitivity analysis was conducted on the included studies, but the source of heterogeneity was not found.

##### Subgroup Analysis

**Figure 6:**
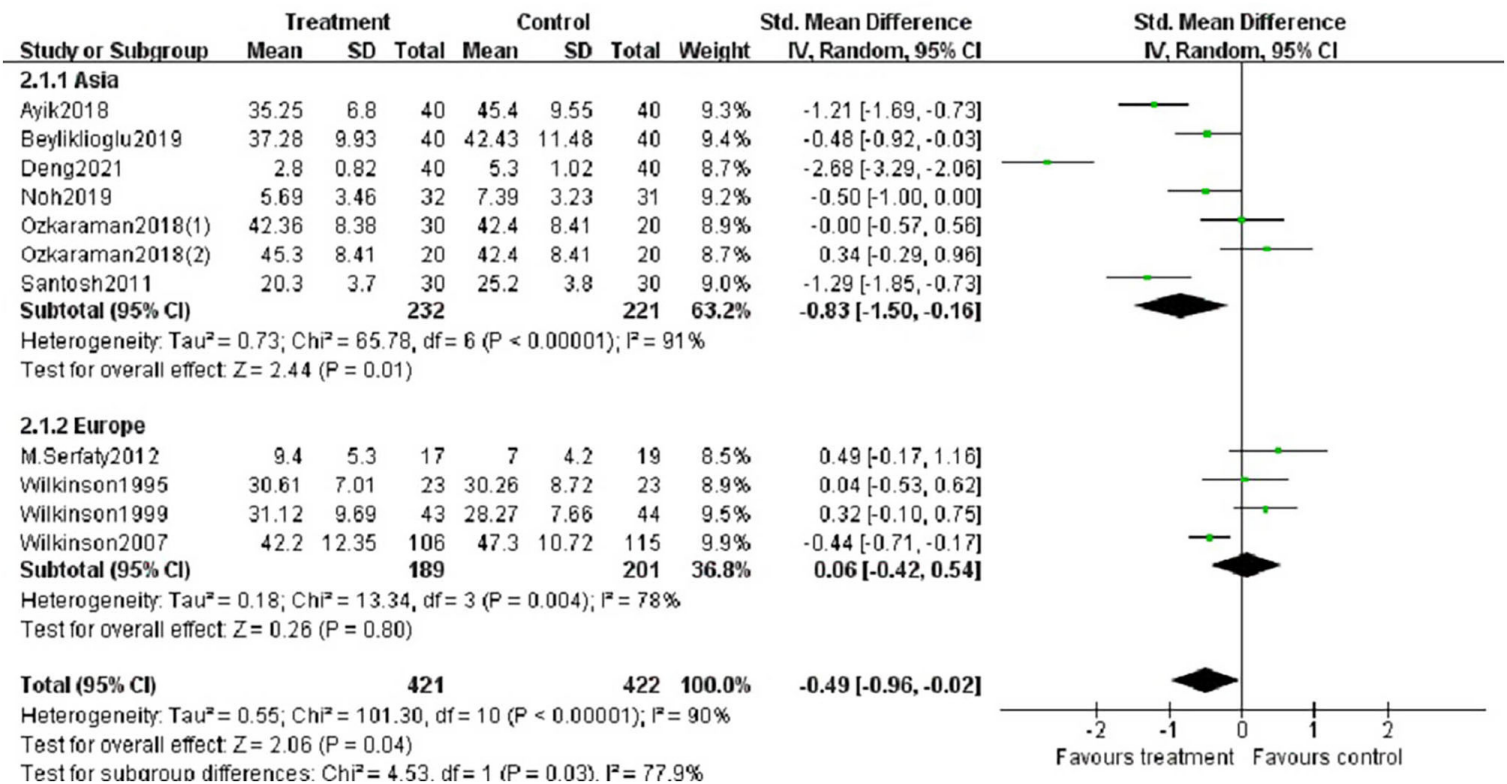
Subgroup analysis according to the study location.

**Figure 7:**
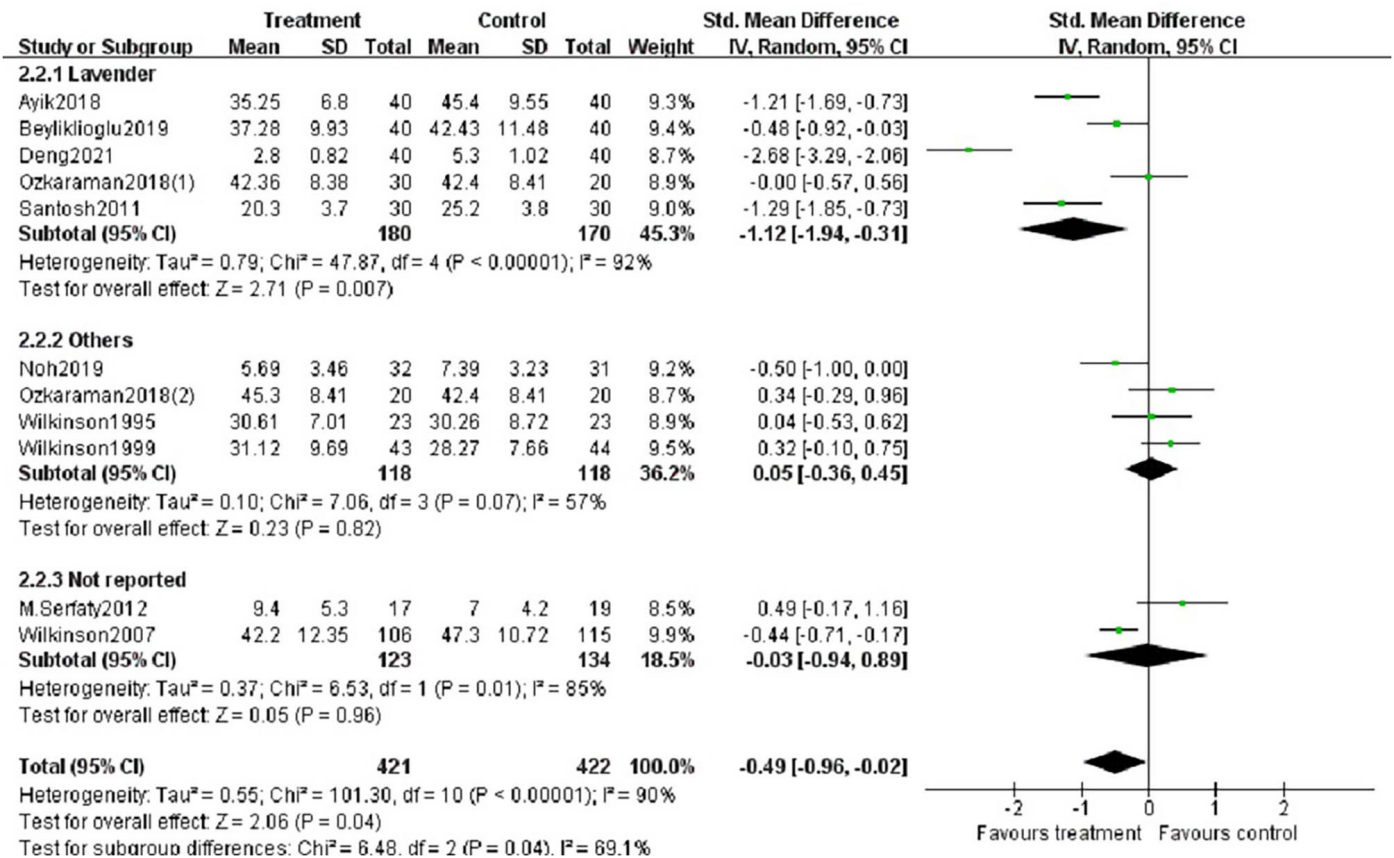
Subgroup analysis according to the type of essential oils.

**Figure 8:**
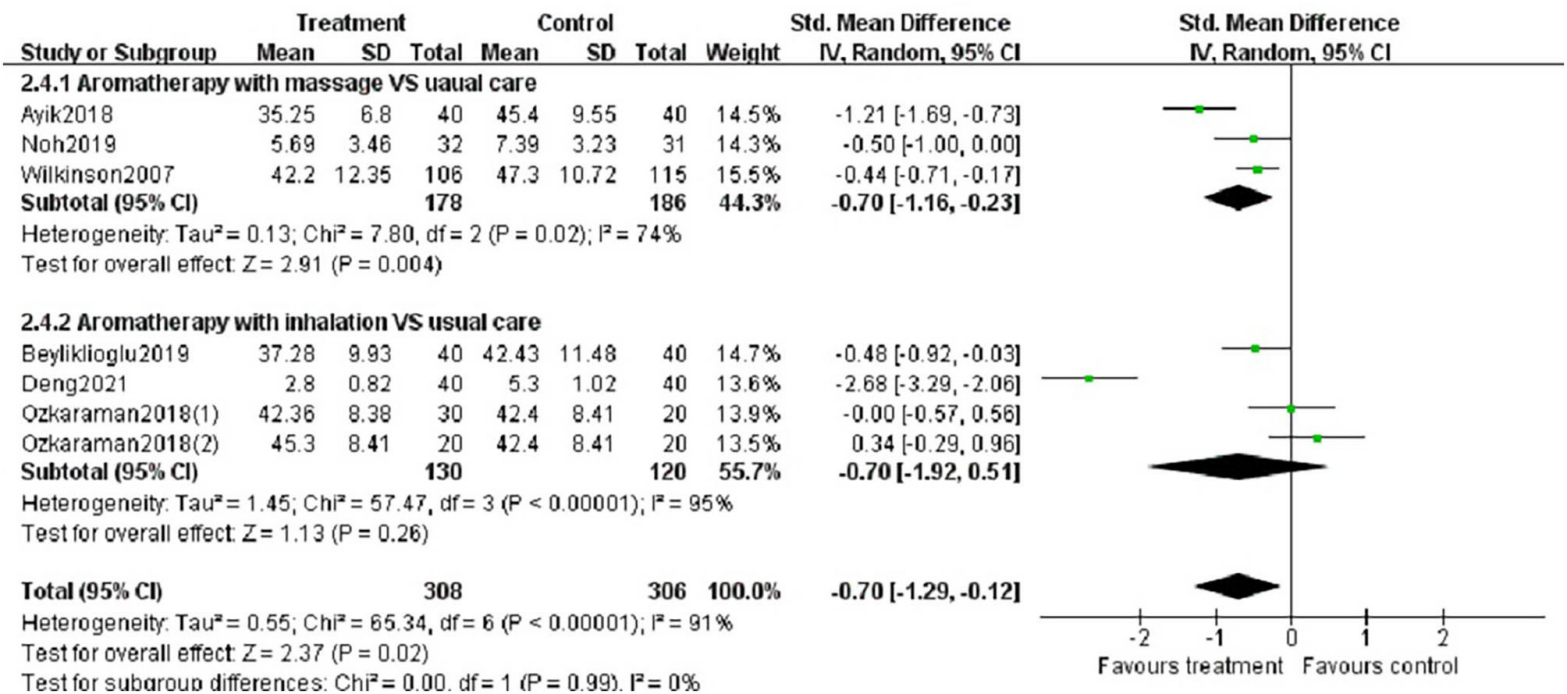
Subgroup analysis according to the type of aromatherapy intervention.

**Figure 9:**
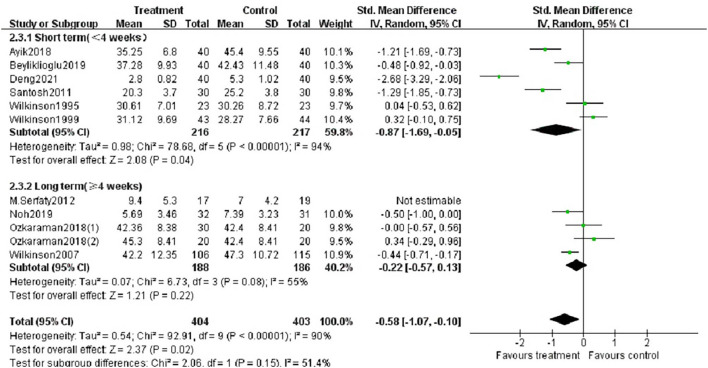
Subgroup analysis according to the duration of aromatherapy intervention.

We performed subgroup analysis based on study location (Asia or Europe), essential oils (lavender, other oils or not reported), type of aromatherapy (massage or inhalation) and duration of intervention (< 4 weeks or ≥ 4 weeks). According to the study location, there was a significant difference between aromatherapy and controls in Asian studies (SMD = −0.83, *p* < 0.05), but not in European studies (Figure 6). The aromatherapy group utilizing lavender essential oils showed a significant effect on reducing anxiety (SMD = −1.12, *p* < 0.01) compared to controls (Figure 7), as the aromatherapy group utilizing massage (SMD = −0.70, *p* < 0.005) (Figure 8). The aromatherapy was significantly effective in reducing anxiety when lasting <4 weeks (SMD = −0.87, *p* < 0.05), but not when lasting longer (Figure 9). Although we concucted subgroup analysis to explore the source of heterogeneity, however, no significant source of heterogeneity was found in the subgroup analysis.

#### Effects of Aromatherapy on Depression

Six studies evaluated the effects of aromatherapy on depression. Five studies utilized aromatherapy massage; a single study used inhaled aromatherapy. The lavender oil was the most used essential oil. Utilizing the HADS scores, Corner et al. described no significant effect of aromatherapy on depression (33). Other two studies found similar findings (36, 43). A single study reported a significant benefit of aromatherapy on depression, compared to controls (*t* = 8.26, *p* < 0.001) (38). According to POMS scores, a study (37) found no significant effect of aromatherapy massage on depression. Another study (47) utilizing the CES-D scale reported no significant effect of aromatherapy massage on depression at 10 weeks post randomization (95% CI, −0.7–5.8; *p* = 0.1).

#### Effects of Aromatherapy on Psychological Wellbeing

Five studies evaluated the effects of aromatherapy on psychological wellbeing. Three studies reported no significant difference between treatment and control groups (43–46). Ovayolu et al. reported a significant difference among the aromatherapy massage group, the aromatherapy inhalation group and the control group (*p* < 0.001) (39). When the POMS scale was used, another study reported no beneficial effect of aromatherapy on psychological wellbeing (44).

## Discussion

### Quality Assessment

The aromatherapy was associated with a high risk of performance bias. Detection bias and attrition bias were also potentially issues. In addition, most of the studies showed unclear allocation concealment. A low risk was observed for selective reporting bias and other bias.

### Effectiveness of Aromatherapy Interventions

Anxiety is an unpleasant emotion of inner nervousness, which could have debilitating consequences for affected individuals (48). Although the measuring tools were different, aromatherapy significantly relieved anxiety in most of the studies. The results were consistent with previous studies performed in women during the first stage of labor (49), patients in hemodialysis therapy (50) and community-dwelling older persons (51). Since the components of essential oils are small lipid soluble molecules, they could be absorbed directly into the bloodstream through the skin or directly through inhalation. The effects of aromatherapy were almost instantaneous and evidence indicated that the effects of essential oils were pharmacological and not just psychological (52). However, Tola et al. did not report any beneficial effect of aromatherapy in women undergoing breast cancer surgery (53). The inconsistency might be due to the fact that we included more studies, performed in people affected by different types of cancer.

Depression is a common medical illness that negatively affects how you feel, the way you think and how you act (54). Only 6 out of 17 studies reported the outcomes of evaluating depression. Due to the heterogeneity between studies, we conducted a systematic review without meta-analysis. Most previous studies found that aromatherapy had no beneficial effect on depression. Only a single study showed that aromatherapy can be a therapeutic option for depressive symptoms (55). Our results were consistent with previous studies (29). The reason why aromatherapy showed no effect on depression may be due to the fact that people with cancer reported more severe depressive symptoms than the general population (56). Moreover, antidepressant therapy required a few months to be effective, while aromatherapy had never been used for more than 10 weeks in the included studies (57).

Only 5 out of 17 studies reported the outcomes of interest about psychological wellbeing. Therefore, we conducted a systematic review without meta-analysis. Most studies showed no beneficial effects of aromatherapy. Our findings were inconsistent with previous studies performed in postmenopausal and elderly women (58). The inconsistency might be related to a number of reasons, including the challenge of correctly measuring psychological wellbeing among different study populations.

### Subgroup Analysis

Regional differences may impact the efficacy of aromatherapy. We found that aromatherapy was effective on anxiety in studies conducted in Asia, but not when studies were conducted in Europe. The cultural context might influence the acceptability and results of an intervention (53). Herbal medicines are traditionally used in Asian countries, people with cancer from these countries might be more receptive to aromatherapy and more likely to believe in its benefits (59). Guo et al. found that using aromatherapy for ≤ 20 min per session was more helpful in reducing anxiety (60). In our study, the Asian group used aromatherapy for a shorter session than the European subgroup, which may partly explain why the European subgroup did not have beneficial effects. In addition, two European studies were conducted several years ago, which makes interpreting them difficult with the most recent studies on the topic.

Regarding the methods of application, aromatherapy massage was significantly effective on anxiety. On the other hand, inhalation aromatherapy showed no beneficial effect. Previous reports described both massage and inhalation as effective in reducing anxiety (61). A meta-analysis conducted in patients with dysmenorrhea showed similar conclusions as ours (28). Aromatherapy massage is a combination of aromatherapy and massage, which provides the health benefits of both approaches (55). The massage itself is a relaxing method designed to relax musecles and to mechanically stimulate the skin and the lymphatic system (62). By increasing parasympathetic activity and decreasing the level of cortisol, massage reduces the level of nervous irritability and increases the level of dopamine and serotonin, which in turn leads to relaxation and mood improvement (63). Furthermore, patients may also inhale small amounts of essential oils (64). This kind of mechanism might combine massage modalities with the stimulation of the olfactory system. The observed effects of massage aromatherapy might be superimposed, it is difficult to distinguish individual effects. In the future, high-quality randomized controlled trials should be designed to investigate the effects of aromatherapy massage and to distinguish individual effects. Although inhaled aromatherapy was not effective in relieving anxiety in our study, standardized trials should be better designed to explore the effects on anxiety because of the advantages of its simplicity and no need for additional labor warrant.

Each essential oil may have specific properties against anxiety and/or depression. The most used essential oil was lavender but only two studies showed lavender essential oil came from Lavandula Hybrida (31, 40). Our results showed the lavender oil was more effective than chamomile oil, mandarin oil, tea tree oil and other rarely used essential oils in our study. Lavender is thought to have the effect of diazepam and the chemical composition has been correlated to their anxiolytic activity (52). Lavender oil is a complex mixture of phytochemicals, including linalool and linalyl acetate (65). Linalool acts as a sedativethat interacts with gamma-aminobutyric acid receptors in the central nervous system (27). After the absorption of lavender oils, the release from the adrenal gland is significantly reduced, while the secretion of serotonin in the digestive system is significantly increased (66). These effects enhances mood, causes sedation and reliefs anxiety. The limbic system might also contribute, providing sedative, relaxing effects and reducing anxiety by interacting with the cerebral cortex and affecting heart rate, blood pressure, respiration, stress, and hormonal levels (67). Many essential oils were used, we did not mention all names and not specify botanical names for each of the essential oils as they were not listed detailedly in the included studies. Due to the limited information, further studies should be carried out to explore the effects of essential oils from specific botanicals on anxiety, and to further clarify the specific effects of different essential oils.

Regarding the treatment duration, we divided the included studies into categories according to the duration of aromatherapy. Interestingly, aromatherapy was effective against anxiety when lasting <4 weeks, but not when it lasted longer. The finding was in accordance with the study of Gong et al. who divided the study subgroups according to the treatment duration (68). The aromatherapy administered for a prolonged period may lead to a decrease in the sensitivity of olfactory receptors, thus decreasing the therapeutic effectiveness (69). Moreover, tumor progression and decline in treatment adherence may explain the lack of efficacy when aromatherapy was applied for more than 4 weeks (70). New studies will explore the real impact of aromatherapy administered for a prolonged period of time.

### Limitations

This study provided a comprehensive overview about the impact of aromatherapy on anxiety or depression in cancer patients. However, this study was afflicted by several limitations. First, we included only studies in English, which limited the generalization of the results. Second, sensitivity analysis and subgroup analysis were conducted, but no source of heterogeneity was detected. Third, blinding was difficult to implement due to the innate features of aromatherapy. Finally, the interpretation of the results required caution because essential oils and measurement tools varied among included studies.

## Conclusion

In conclusion, aromatherapy might be an effective therapeutic option in alleviating anxiety for cancer patients. The therapy was particularly effective when administered through massage or with the use of lavender oil, for <4 weeks or in studies conducted in Asia. However, aromatherapy showed no beneficial effect on depression and psychological wellbeing. The methods of intervention were different, thus future studies with more standardized protocols need to be devised.

## Data Availability Statement

The original contributions presented in the study are included in the article/Supplementary Material, further inquiries can be directed to the corresponding author.

## Author Contributions

CYJ was responsible for the study design. LD drafted the manuscript. LD, LYX, BX, and WMJ developed search strategies, screened articles, assessed the quality of articles, and extracted the data. YJZ participated in the statistical analysis. All authors read and approved the final manuscript.

## Funding

This research was funded by the Chinese Association for Life Care.

## Conflict of Interest

The authors declare that the research was conducted in the absence of any commercial or financial relationships that could be construed as a potential conflict of interest.

## Publisher's Note

All claims expressed in this article are solely those of the authors and do not necessarily represent those of their affiliated organizations, or those of the publisher, the editors and the reviewers. Any product that may be evaluated in this article, or claim that may be made by its manufacturer, is not guaranteed or endorsed by the publisher.
