# Supplementary material for: The Effects of Aromatherapy on Anxiety and Depression in People With Cancer: A Systematic Review and Meta-Analysis
Source: Front Public Health. 2022 May 30;10:853056. doi: 10.3389/fpubh.2022.853056 (PMC9215260; doi:10.3389/fpubh.2022.853056)
Supplement: Supplementary file 2 [file Table_2.DOCX]

Supplementary Table 2. Search strategies used for searching the databases

| Cochrane Library | aromatherapy OR fragrance OR (essential oil) OR (scent therapy) OR (aroma therapy) in Title Abstract Keyword  AND anxi* OR depress* OR emotion* OR psycholog* OR disorder* in Title Abstract Keyword  AND neoplas* OR cancer OR tumor OR carcinoma OR malignancy in Title Abstract Keyword |
| --- | --- |
| Ovid-medline | aromatherapy or fragrance or “essential oil” or “scent therapy” or “aroma therapy”.mp.  AND anxi* or depress* or emotion* or psycholog* or disorder* .mp.  AND neoplas* or cancer or tumor or tumour or carcinoma or malignancy .mp. |
| ProQuest | noft (anxi* or depress* or emotion* or psycholog* or disorder*)  AND noft (aromatherapy or fragrance or “essential oil” or “scent therapy” or “aroma therapy”)  AND noft (neoplas* or cancer or tumor or tumour or carcinoma or malignancy) |
| EbscoHost | AB (aromatherapy or fragrance or “essential oil” or “scent therapy” or “aroma therapy”)  AND AB ( anxi* or depress* or emotion* or psycholog* or disorder* )  AND AB (neoplas* or cancer or tumor or tumour or carcinoma or malignancy) |
| Scopus | Title-abs-key (aromatherapy or fragrance or “essential oil” or “scent therapy” or “aroma therapy”)  AND Title-abs-key ( anxi* or depress* or emotion* or psycholog* or disorder* )  AND Title-abs-key ( neoplas* or cancer or tumor or tumour or carcinoma or malignancy) |
| EMBASE | aromatherapy OR fragrance OR (essential oil) OR (scent therapy) OR (aroma therapy)  AND anxi* OR depress* OR emotion* OR psycholog* OR disorder*  AND neoplas* or cancer or tumor or tumour or carcinoma or malignancy |
| Web of science | Title aromatherapy OR fragrance OR (essential oil) OR (scent therapy) OR (aroma therapy)  AND Theme anxi* OR depress* OR emotion* OR psycholog* OR disorder*  AND Theme neoplas* OR cancer OR tumor OR carcinoma OR malignancy |
